# Risk factor analysis of *Campylobacter* spp., *Listeria monocytogenes* and *Salmonella* spp. in the chicken meat value chain

**DOI:** 10.3389/fmicb.2025.1750419

**Published:** 2026-01-12

**Authors:** Elisetty Naga Pavana Sneha, Ekkoruparambil Sethurajan Sanjumon, Hung Nguyen-Viet, Zunjar Baburao Dubal, Kiran Narayan Bhilegaonkar, Haris Ayoub, Delia Grace Randolph, Bhuva Akash, Amit Gangwar, Sukanna Manoj, Dharavath Premkumar, Himani Dhanze, Bablu Kumar, Obli Rajendran Vinodh Kumar, Murthy Suman Kumar, Ram Pratim Deka

**Affiliations:** 1 Division of Veterinary Public Health, ICAR-Indian Veterinary Research Institute, Izatnagar, India; 2 International Livestock Research Institute, Nairobi, Kenya; 3 International Livestock Research Institute, National Agricultural Science Complex, New Delhi, India; 4 Natural Resources Institute, University of Greenwich, Chatham, United Kingdom; 5 Division of Epidemiology, ICAR-Indian Veterinary Research Institute, Izatnagar, India

**Keywords:** *L. monocytogenes*, *Campylobacter* spp., chicken meat value chain, food safety, risk factors, *Salmonella* spp.

## Abstract

**Introduction:**

A cross-sectional study was conducted to evaluate microbiological risk factors associated with *Salmonella* spp., *Campylobacter* spp., and *Listeria monocytogenes* along the chicken meat value chain in Bareilly district, Uttar Pradesh, India.

**Methods:**

Following multistage cluster sampling design a total of 941 samples from three nodes (retailers, restaurants, consumer households) were collected. The retailers’ samples (*n* = 519) included raw meat (*n* = 360), water (*n* = 83) and swabs (*n* = 76). The restaurants samples (*n* = 242) and consumer households (*n* = 180) were cooked chicken meat samples. Isolation followed ISO-based culture methods with PCR confirmation; presence of any target pathogen was combined into a binary “pathogen indicator.” A structured questionnaire was used to collect information regarding the practices of the retailers (*n* = 127), restaurant owners (*n* = 101) and chicken meat consumers (*n* = 180). Univariate analysis was performed between the factors and presence of pathogen indicator. The factors with *p* < 0.2 were used in multivariable logistic regression to identify independent predictors of contamination at each node.

**Results:**

At the retail level, significant risk factors included unclean costumes (OR = 2.130, 95% CI, 1.348–3.367) and selling chicken meat in open space (OR = 1.675, 95% CI, 1.063–2.640). Conversely, trimmed nails (OR = 0.247, 95% CI, 0.154–0.398) and using glass covers (OR = 0.636, 95% CI, 0.404–1.00) for retail outlets were protective factors. At the restaurant level, using raw vegetables as garnish (OR = 4.257, 95% CI, 1.181–15.345) had significantly higher odds of pathogen presence. Protective factors included using separate cutting boards or knives (OR = 0.153, 95% CI, 0.052–0.447) and keeping bulk-cooked products hot (OR = 0.322, 95% CI, 0.113–0.920). At the consumer level, washing hands only before handling (OR = 12.60, 95% CI, 3.124–50.82) was found to be a significant risk factor. Protective factors included using separate cutting boards/knives for raw meat and vegetables (OR = 0.067, 95% CI, 0.017–0.262) and adding raw vegetables during cooking instead of adding after cooking (OR = 0.175, 95% CI, 0.052–0.585).

**Discussion:**

Findings indicate that retail-stage infrastructure and hygiene practices are primary drivers of contamination, and that pragmatic interventions are likely to reduce downstream foodborne risk.

## 1 Introduction

Foodborne diseases are a significant global health issue, leading to considerable morbidity and mortality (WHO, 2015). These illnesses can arise at any point along the food production, distribution, or consumption chain and persist in both high- and low-income countries (Grace, 2023). In Low- and Middle-Income Countries (LMICs), food safety challenges are exacerbated by the inherent structure of the food sector. These systems are typically fragmented and diverse, characterized by a vast network of small-scale operators and a dominant informal sector that operates with minimal organizational cohesion (Grace, 2015). Contamination may result from multiple sources, including water, air, and soil, and is often aggravated by unsafe handling, improper storage, and inadequate processing. Globally, about 10% of the population suffers from foodborne illnesses annually, leading to over 420,000 deaths (WHO, 2015). In 2010, contaminated food caused approximately 600 million cases and 33 million disability-adjusted life years (DALYs), affecting nearly one in ten people worldwide (Havelaar et al., 2015).

Foods of animal origin, particularly meat, milk, eggs, and their products, are major contributors to foodborne infections. In India, meat production has steadily increased, reaching 9.77 million tons in 2022–23 (Department of Animal Husbandry and Dairying, 2023), with poultry meat constituting about 51.14% of total output. Affordability and widespread acceptance among all communities are the key factors driving the increased consumption of eggs and poultry meat (Mohanapriya et al., 2023). It is preferred for its high protein content, low fat, affordability, and short rearing cycles (Karabasanavar et al., 2020). The rise in per capita meat consumption, from 6.15 kg in 2018–19 to 7.39 kg in 2023–24, is primarily driven by the expansion of the poultry sector. As poultry meat already accounts for a significant share of the country’s total meat output, its increased production—combined with a growing public awareness of protein-rich diets—has directly fueled this (Department of Animal Husbandry and Dairying, 2025). However, the poultry sector faces major food safety and hygiene challenges, as most meat is sold through informal wet markets lacking proper infrastructure and cold chain systems (Gulati and Juneja, 2023). Such deficiencies may increase the likelihood of contamination by microorganisms, including bacteria, viruses, fungi, and parasites, that can cause food poisoning.

Globally, about two-thirds of foodborne disease outbreaks occur in homes and catering establishments (Hedberg et al., 2006), which are particularly vulnerable due to large meal volumes and extensive handling. Foodborne pathogens may be introduced at multiple stages, from raw material production, storage to processing and distribution, with key sources including animals, the environment, and human handlers (Bridier et al., 2015). Common risk factors include unsafe food sources, inadequate cooking, improper holding temperatures, and contaminated equipment (WHO, 2023). Slaughtering transfers bacteria from the animal and equipment onto the meat. This contamination can persist, and sometimes even grow, on the final cuts during processing and storage (Rouger et al., 2017). Although a healthy bird’s muscle tissue is sterile, its skin, feathers, and internal tracts naturally carry diverse microbes. The slaughterhouse itself—including its surfaces, air, and water—also contains bacteria. During processing, these two sources combine, leading to the contamination of carcasses and meat cuts by microbes from both the animal and the environment (Rouger et al., 2017).

*Salmonella* spp., *Campylobacter* spp., and *Listeria monocytogenes* are three critical foodborne pathogens associated with poultry. Non-typhoidal *Salmonella* (NTS) is a major cause of illness, resulting in 93.8 million cases and 155,000 deaths annually (Majowicz et al., 2010), with *Salmonella Enteritidis* and *Salmonella Typhimurium* being predominant globally (Galanis et al., 2006; Reddy et al., 2010). Recent surveillance across India highlights a significant, albeit geographically fragmented, burden of foodborne pathogens within the poultry value chain. The prevalence of *Salmonella* spp. in retail chicken meat demonstrates extreme regional heterogeneity, ranging from hyper-endemic levels of 74% in Mumbai (Shashidhar et al., 2011) and 47% in the Delhi-Haryana belt (Bhardwaj et al., 2022) to a comparatively low 4.83% in Karnataka (Karabasanavar et al., 2020). Among foodborne transmission routes, the consumption of raw or undercooked chicken meat is recognized as the single largest potential source of human *Campylobacter* infection (Popa et al., 2022). Campylobacteriosis, primarily from *Campylobacter jejuni* and *Campylobacter coli*, is a high-burden disease strongly linked to poultry (Abebe et al., 2020), which carries high intestinal loads (10^6^–10^8^ CFU/g) (Oh et al., 2018), and can infect humans at low doses, sometimes leading to severe complications like Guillain-Barré syndrome (Poropatich et al., 2010). *Campylobacter* spp. isolation rates vary across the Indian subcontinent, with high prevalence reported in West Bengal (72%) (Sharma et al., 2016) and Northern India (38.6%) (Khan et al., 2018), in sharp contrast to the 2.3% incidence observed in broiler meat samples (Kumar et al., 2023). *L. monocytogenes*, while less prevalent, is highly severe, with 20–30% mortality and 91% hospitalization (Jemmi and Stephan, 2006; Dhama et al., 2015). In India its distribution is notably erratic; isolation rates vary from 16% in Chhattisgarh (Beigh et al., 2019) and 8.5% in North East India (Shakuntala et al., 2019) to 1.7% in Punjab (Kaur et al., 2018), with complete absence (0%) reported in Kerala (Latha et al., 2017). It poses a unique threat in the poultry value chain due to its ability to survive refrigeration and form biofilms in processing environments (Colagiorgi et al., 2017), leading to severe outcomes like meningitis in vulnerable populations (Mateus et al., 2013; Jamshidi and Zeinali, 2019). A recent PRISMA-based systematic review (2010–2023, 90 studies) highlighted that Indian retail poultry meat frequently carries major foodborne pathogens, with *Campylobacter* spp. and *Salmonella* spp. each showing a pooled prevalence of 18% each (95% CI, 11–27% and 11–26%, respectively), while *L. monocytogenes* was also detected at 13% (95% CI, 1–33%). The review further underscored that *Campylobacter* and *Salmonella* isolates exhibited high resistance to critically important antimicrobials, including erythromycin, tetracycline, ciprofloxacin, and colistin, posing a substantial public-health concern (Ayoub et al., 2025). Despite this burden, studies from India that examine upstream or downstream risk factors in poultry production, retail, or consumer handling remain scarce. These findings reinforce the importance of formal surveillance of Indian poultry systems, to better understand contamination patterns and associated risk factors for the priority pathogens.

The present study was conducted to identify the risk factor associated with the microbiological quality of chicken meat at chicken meat value chain with a specific focus on pathogen indicators such as *Salmonella* spp., *Campylobacter* spp., and *L. monocytogenes*. Identification of the risk factors associated with the pathogen indicators in the chicken meat value chain may give insight to take necessary steps to reduce microbial contamination.

## 2 Materials and methods

### 2.1 Sampling and data collection

The study was conducted in Bareilly district of Uttar Pradesh in India situated at 28.5426° N and 79.4704° E. Bareilly district has six Tehsils, 15 blocks and 2070 villages with a population of 44,48,359 (Directorate of Census Operations, Uttar Pradesh, 2011) and literacy rate of 58.49%. In the Bareilly district, the chicken meat value chain comprises several key actors, including producers, dealers, retailers, consumers, and restaurants. Three Tehsils out of six in the Bareilly district were selected by simple random sampling using online random number generator. From these three Tehsils, four blocks were selected viz.: Baheri, Bareilly, Bithrichainpur and Nawabganj. Consequently, from each block, clusters consisting of chicken value chain actors viz., Chicken Meat Retailers, Chicken Meat Consumers and Restaurant Owners were identified with the help of field visit. The representative clusters were identified for sampling by random selection wherein each cluster had all three chicken value chain actors. A 50% prevalence was considered for each pathogen to get the maximum sample size. Intra-cluster correlation was assumed to be less (0.03), and the margin of error was fixed at 5% with 80% power and 95% confidence level. Estimating a design effect of 2.2 for the sampling method, the effective sample size for the design was calculated as 834. Further, assuming 10% of the allowance of sample spoilage/non-respondents of interview, the final sample size was fixed at 930.

From June 2024 to November 2024, raw meat (*n* = 360) as well as water (*n* = 83) and swab (*n* = 76) samples were collected from Chicken Meat Retailers and cooked meat samples were collected from Restaurants (*n* = 242) and Consumer households (*n* = 180) (Table 1). Multiple raw chicken meat samples were taken from retailers at different points of time. Similarly, different types of cooked chicken meat samples were collected from same restaurants (Table 2). Separate peer reviewed questionnaires were prepared for each value chain actor with the help of observations from a stakeholder meeting and Key informants interview as well as opinions from experts in the field. This has been validated by field testing in a small sample population consisting of targeted value chain actors. Data on microbiological risk factors were collected from key value chain actors using this field-tested structured questionnaires. Questionnaires were administered to 127 chicken meat retailers, 101 restaurant owners, and 180 consumers. These instruments captured information on practices influencing microbial quality, including hygiene, handling, storage, preparation, cooking methods, and reheating. All responses were subsequently cleaned and compiled in Microsoft Excel for statistical analysis.

**Table 1:** Block-wise sample collected from each value chain actor.

Sl. No.	Chicken value chain actor	Number of actors from each block				Total
		Baheri	Bareilly	Bithrichainpur	Nawabganj	
1	Retailer	30	30	30	37	127
2	Restaurant	31	45	14	11	101
3	Consumer	52	18	55	55	180
Total		113	93	99	103	408

**Table 2:** Actor-wise samples collected from each block.

Sl. No.	Chicken value chain actor	Type of sample	Number of samples from each block				Total
			Baheri	Bareilly	Bithrichainpur	Nawabganj	
1	Retailer	Raw meat	93	74	87	106	360
		Water	19	10	27	27	83
		Swab	18	12	21	25	76
2	Restaurant	Cooked meat	60	135	33	14	242
3	Consumer	Cooked meat	52	18	55	55	180
Total			242	249	223	227	941

### 2.2 Laboratory analysis

A total of 25 g each of raw and cooked meat, associated retail outlet swabs collected in sterile normal saline, and 250 mL of water used in the outlet were collected and processed from these value chain actors at Veterinary Public Health division of IVRI within 24 h of collection. For *Campylobacter* spp. (ISO 10272-1), samples were enriched in Preston broth and plated on modified charcoal cefoperazone deoxycholate (mCCD) agar, incubated under microaerophilic conditions, with typical colonies confirmed by PCR targeting the *16S rRNA* gene (Linton et al., 1996). *C. jejuni* ATCC 33291 was utilized as a positive control in all PCR assays. For *Salmonella* spp. (ISO 6579-1), enrichment was performed in Buffered Peptone Water (BPW), Rappaport-Vassiliadis soya (RVS) broth, and Muller-Kauffmann tetrathionate-novobiocin (MKTTn) broth, followed by plating on Xylose Lysin Deoxycholate (XLD) agar and Brilliant Green (BGA) agar, with typical colonies sub-cultured and confirmed by PCR for the *invA* gene (Chiu and Ou, 1996). *S. Typhimurium* ATCC 13311 was utilized as a positive control in all PCR assays. Finally, for *L. monocytogenes* (ISO 11290-1), samples were enriched in Half Frasier and Frasier broths, plated on Agar Listeria Ottovani Agosti agar (ALOA), Polymyxin Acriflavin Lithium-chloride Ceftazidime Esculin Mannitol (PALCAM) agar and confirmed via PCR targeting the *prs* and *isp* genes (Rawool et al., 2016). *L. monocytogenes* ATCC 19114 was used as positive control in relevant reactions. For all the PCR assays, Nuclease-free Water was used as template in negative control.

### 2.3 Risk factor analysis

The data obtained from microbiological analysis of collected samples were mapped against the respective value chain actors from where the samples were collected. For effective analysis and interpretation, all three pathogens (*Salmonella* spp., *L. monocytogenes* and *Campylobacter* spp.) were combined and considered as a single “pathogen indicator” to represent overall food safety risk. The presence of “pathogen indicator” was coded as ‘1’ and absence was coded as ‘0’ in the excel sheet. The potential risk factors considered as detailed in questionnaire were taken as independent variables and presence of ‘pathogen indicator’ as response variable for statistical analysis. Univariate and multivariable analysis were performed in R statistical platform (Ver. 4.5) with appropriate packages. Cross-tabulation or Fisher’s exact test was used to test the significance of the associations between the explanatory variables and the outcome variable. Fisher’s exact test was used when expected cell frequencies were <5. Analysis of multiple predictors of positivity was performed using stepwise forward logistic regression analysis considering only those factors significant at *p* ≤ 0.2 in Univariate analysis and retaining only factors significant at *p* ≤ 0.05 in the final model (VinodhKumar et al., 2020).

## 3 Results

The combined prevalence of the pathogen indicator was 30.0% (108/360) in raw chicken meat at the retailer level, 6.7% (12/180) in consumer-cooked meat, and 6.6% (16/242) in restaurant-cooked meat (Table 3). Among the chicken meat samples examined, *Salmonella* spp. was the most prevalent, being isolated from 23.3% (84/360) of raw meat samples at the retailer level, 6.7% (12/180) of consumer-cooked meat samples, and 6.2% (15/242) of restaurant-cooked meat samples. *Campylobacter* spp. was detected in 9.4% (34/360) of raw meat samples collected from retailers, whereas only 0.6% (1/180) of consumer-level cooked meat samples and 0.4% (1/242) of restaurant-cooked meat samples tested positive. *L. monocytogenes* was detected in 0.6% (2/360) of retailer-level raw meat samples, while none of the consumer or restaurant-cooked meat samples showed its presence.

**Table 3:** Sample-wise prevalence of pathogen indicator.

Sl. No.	Value chain actor	Sample type	Number of positive samples	Total number of samples	Prevalence (%)	95 C. I.
1	Retailer	Raw meat	108	360	30	25–35
		Water	9	83	10.84	5.6–19.5
		Swab	7	76	9.21	4.2–18.1
2	Restaurant	Cooked meat	16	242	6.61	4.4–10.5
3	Consumer	Cooked meat	12	180	6.67	3.7–11.4
Total			152	941	16.15	13.9–18.6

### 3.1 Risk factor analysis

#### 3.1.1 Retail level

A pre-slaughter holding period of more than 24 hours was a significant protective factor with lower odds of contamination (OR = 0.311, 95% CI, 0.102–0.946) compared to when birds were slaughtered within 2 h of stocking in retail outlet. Slaughtering and selling interval of 31–60 min was found to be a significant risk factor for pathogen presence (OR = 14.902, 95% CI, 5.853–37.941) compared to the reference category of meat sold within 10 min of slaughter. Washing the retail outlet every 4–5 h was a significant protective factor with a markedly reduced odds of pathogen presence (OR = 0.140, 95% CI, 0.084–0.232) compared to washing time interval of 10–12 h. Other significant risk factors for presence of pathogen indicator at retail level includes, lack of cleanliness of costumes (OR = 2.130, 95% CI, 1.348–3.367), presence of high number of flies in and around the retail outlet (OR = 3.0, 95% CI, 1.841–4.889), selling chicken meat in open space (OR = 1.675, 95% CI, 1.063–2.640), and lack of glass covers to prevent dust (OR = 0.636, 95% CI, 0.404–1.00).

Trimmed nails (OR = 0.247; 95% CI: 0.154–0.398), covering meat with fly nets (OR = 0.389; 95% CI: 0.241–0.627), and the absence of hand jewelry (OR = 0.535; 95% CI: 0.337–0.849) were identified as significant protective factors. Each of these hygiene practices were associated with significantly reduced odds of pathogen presence.

Multivariable logistic regression identified several independent predictors of contamination. Significant protective factors included a pre-slaughter holding period of 6.1–12 h (AOR = 0.232, 95% CI, 0.055–0.979), trimmed nails (AOR = 0.193, 95% CI, 0.089–0.421), and outlet washing intervals of 4–5 h (AOR = 0.051, 95% CI, 0.020–0.130) and >24 h (AOR = 0.037, 95% CI, 0.004–0.322) relative to the 10–12 h reference. Conversely, a slaughter-to-sale interval of 31–60 min (AOR = 38.079, 95% CI, 9.555–151.755), visible fly density (AOR = 3.898, 95% CI, 1.697–8.952) and selling chicken meat in open space (AOR = 2.621, 95% CI, 1.184–5.804) were associated with increased odds of pathogen presence (Tables 4, 5). The −2 log likelihood value of the model was obtained as 213.207 and the Hosmer and Lemeshow test was not significant.

**Table 4:** Univariate analysis of pathogen indicator risk factors at retailer level.

Risk factors	Categories	OR (95%CI)	*p* value
Time period between buying live birds and slaughtering them	<2 h	Ref	
	>24 h	0.311 (0.102–0.946)	0.040
	12.1–24 h	0.444 (0.161–1.224)	0.117
	2–6 h	0.822 (0.356–1.895)	0.646
	6.1–12 h	0.440 (0.189–1.025)	0.057
Time period between slaughtering and selling	<10 min	Ref	
	1–3 h	2.751 (0.794–9.528)	0.110
	10–30 min	0.332 (0.185–0.597)	0.000
	3–6 h	1.146 (0.101–12.999)	0.912
	31–60 min	14.902 (5.853–37.941)	0.000
Storage of meat after slaughter if not sold immediately	Outside covered with cotton	Ref	
	Outside in vessel with fly net	0.389 (0.241–0.627)	0.000
	Outside in vessel without fly net	0.904 (0.146–5.582)	0.913
	Other arrangements to keep meat cool	0.417 (0.129–1.344)	0.143
	Refrigerator	0.000 (0.000)	0.999
Type of surface on which meat was kept after slaughter	Concrete	Ref	
	Plastic	2.249 (0.611–8.275)	0.223
	Tile or marble	1.367 (0.367–5.086)	0.641
	Wooden plank	3.000 (0.805–11.178)	0.102
Cleanliness of costumes	Clean	Ref	0.001
	Unclean	2.130 (1.348–3.367)	
Trimmed nails	No	Ref	0.000
	Yes	0.247 (0.154–0.398)	
Ornaments in fingers or hands	Yes	Ref	0.008
	No	0.535 (0.337–0.849)	
Presence of glass covering	No	Ref	0.050
	Yes	0.636 (0.404–1.001)	
Time since the retail outlet was last washed to sampling	10–12 h ago	Ref	
	4–5 h ago	0.140 (0.084–0.232)	0.000
	>24 h ago	0.303 (0.077–1.193)	0.088
Visible fly density	No	Ref	0.000
	Yes	3.00 (1.841–4.889)	
Selling chicken meat in open space	No	Ref	0.025
	Yes	1.675 (1.063–2.640)	

Ref, Reference category.

**Table 5:** Multivariable binary logistic regression—retailer level.

Risk factors	Categories	AOR (95%CI)	*P* value
Time period between buying live birds and slaughtering them	<2 h	Ref	
	>24 h	0.255 (0.044–1.479)	0.128
	12.1–24 h	0.317 (0.064–1.583)	0.161
	2–6 h	2.760 (0.656–11.611)	0.166
	6.1–12 h	0.232 (0.055–0.979)	0.047
Time period between slaughtering and selling	<10 min	Ref	
	1–3 h	9.678 (1.198–78.170)	0.033
	10–30 min	0.207 (0.088–0.486)	0.000
	3–6 h	0.470 (0.023–9.623)	0.624
	31–60 min	38.079 (9.555–151.755)	0.000
Storage of meat after slaughter if not sold immediately	Outside covered with cotton	Ref	
	Outside in vessel with fly net	0.710 (0.315–1.600)	0.408
	No fly net	0.473 (0.006–35.232)	0.733
	Other arrangements to keep meat cool	0.666 (0.123–3.599)	0.637
	Refrigerator	0.000 (0.000)	0.999
Type of surface on which meat was kept after slaughter	Concrete	Ref	
	Plastic	5.103 (0.519–50.205)	0.162
	Tile or marble	10.493 (0.963–114.319)	0.054
	Wooden plank	4.316 (0.427–43.623)	0.215
Personnel hygiene indicators	Cleanliness of costumes	0.785 (0.363–1.698)	0.538
Personnel hygiene indicators	Trimmed nails	0.193 (0.089–0.421)	0.000
Personnel hygiene indicators	Ornaments in fingers or hands	0.601 (0.288–1.257)	0.176
Outlet cleanliness	Presence of glass covering	0.580 (0.269–1.253)	0.166
Outlet cleanliness	Visible fly density	3.898 (1.697–8.952)	0.001
Outlet cleanliness	10–12 h since the retail outlet was last washed to sampling	Ref	
	4–5 h since the retail outlet was last washed to sampling ago	0.051 (0.020–0.130)	0.000
	>24 h since the retail outlet was last washed to sampling	0.037 (0.004–0.322)	0.003
Outlet cleanliness	Using disinfectants for cleaning floors and surfaces	1.382 (0.542–3.521)	0.498
Outlet cleanliness	Selling chicken meat in open space	2.621 (1.184–5.804)	0.018

Ref, Reference category.

#### 3.1.2 Restaurant level

At restaurant level, garnishing the cooked meat product with raw vegetables (OR = 4.257, 95% CI, 1.181–15.345) as well as keeping raw, preheated and heated products together in fridge (OR = 3.388, 95% CI, 1.155–9.936) were associated with higher odds of contamination. Using separate cutting boards or knives for raw meat and vegetables (OR = 0.153, 95%CI, 0.052–0.447), keeping the bulk-cooked meat products hot (OR = 0.322, 95% CI, 0.113–0.920) and bulk cooking of the meat products (OR = 0.248, 95% CI, 0.086–0.709) came out as protective factors with significant lower odds of contamination. Interestingly, cooking multiple products simultaneously (OR = 0.201, 95% CI, 0.063–0.643) also was having lower odds of contamination.

The final adjusted Multivariable model found two independent predictors. Bulk cooking (AOR = 0.076, 95% CI, 0.015–0.374), and cooking multiple products simultaneously (AOR = 0.177, 95% CI, 0.037–0.846) were found to have significant lower odds of pathogen presence (Tables 6, 7). The −2 log likelihood value of the model was obtained as 74.019 and the Hosmer and Lemeshow test was not significant.

**Table 6:** Univariate analysis of pathogen indicator risk factors at restaurant level.

Factors	Categories	OR (95%CI)	*p* value
Use of separate cutting boards/knives for raw meat and vegetables	No	Ref	0.000
	Yes	0.153 (0.052–0.447)	
Cooking the product in bulk	No	Ref	0.006
	Yes	0.248 (0.086–0.709)	
Keeping bulk cooked product hot after preparation	No	Ref	0.027
	Yes	0.322 (0.113–0.920)	
Using vegetables as garnish	No	Ref	0.017
	Yes	4.257 (1.181–15.345)	
Keeping raw, preheated and heated products together in fridge	No	Ref	0.019
	Yes	3.388 (1.155–9.936)	
Cooking more than one type of products at the same time	No	Ref	0.003
	Yes	0.201 (0.063–0.643)	

Ref, Reference category.

**Table 7:** Multivariable binary logistic regression—restaurant level.

Risk factors	Categories	OR (95%CI)	*P* value
Cooking method followed	Biryani	Ref	
	Boiling	0.679 (0.136–3.376)	0.636
	Frying	0.188 (0.028–1.257)	0.085
	Grilled/roasted	0.547 (0.056–5.320)	0.603	
	Using separate cutting boards and knives used for raw chicken meat and vegetables	0.364 (0.110–1.210)	0.099
	Wearing hair cap during handling and preparation of the product	0.436 (0.113–1.686)	0.229
	Cooking the product in bulk	0.076 (0.015–0.374)	0.002
	Keeping cooked bulk product hot after preparation	0.730 (0.217–2.454)	0.611
	Using raw vegetables as garnish	3.414 (0.862–13.526)	0.080
	Keeping raw, preheated, and cooked meat together in the refrigerator	2.981 (0.695–12.782)	0.141
	Cooked more than one type of product at the same time	0.177 (0.037–0.846)	0.030

Ref, Reference category.

#### 3.1.3 Consumer level

At consumer level, washing hands only before handling the raw meat was having significantly higher odds (OR = 12.60, 95% CI, 3.124–50.82) compared to washing before and after handling. Similarly, reheating the meat dishes instead of cooking and eating fresh was also associated with higher odds (OR = 6.164, 95% CI, 1.605–23.676) for pathogen presence. Adding raw vegetables during cooking (OR = 0.175, 95% CI, 0.052–0.585) compared to adding it at the end of cooking and using separate cutting board or knives for raw meat and vegetables (OR = 0.067, 95% CI, 0.017–0.262) were significant protective factors with significant lower odds for pathogen presence.

In multivariable binary logistic regression, washing hands only before handling raw meat (AOR = 10.891, 95% CI, 1.768–67.068) and using separate cutting boards and knives (AOR = 0.079, 95% CI, 0.013–0.494) were found to have significant lower odds of contamination (Tables 8, 9). The −2 log likelihood value of the model was obtained as 51.088 and the Hosmer and Lemeshow test was not significant.

**Table 8:** Univariate analysis of pathogen indicator risk factors at consumer level.

Risk	Categories	OR (95%CI)	*p* value
Washing hands before and after handling raw meat	Always before and after	Ref	
	Only after	0.000 (0.000)	0.997
	Only before	12.600 (3.124–50.824)	0.000
Time of adding raw vegetables to cooked meat	At the end of cooking	Ref	0.002
	While cooking	0.175 (0.052–0.585)	
Using separate cutting board/ knives for cutting raw meat and vegetables	No	Ref	0.000
	Yes	0.067 (0.017–0.262)	
Whether dishes are reheated and consumed	Fresh	Ref	0.003
	Reheated	6.164 (1.605–23.676)	

Ref, Reference category.

**Table 9:** Multivariable binary logistic regression—consumer level.

Risk factors	Category	OR	*p*
Were hands washed before and after handling raw meat	Always before and after	Ref	
	Only after	0.000 (0.000)	0.997
	Only before	10.891 (1.768–67.068)	0.010	
	Adding vegetables during preparation of the dish	7.163 (0.704–72.864)	0.096
	Using separate cutting boards and knives for raw chicken meat and vegetables	0.079 (0.013—0.494)	0.007
	Freshly cooking the chicken dish	5.409 (0.669–43.745)	0.113

Ref, Reference category.

## 4 Discussion

The present study was conducted to identify the risk factor associated with the microbiological quality of chicken meat at chicken meat value chain with a specific focus on *Salmonella* spp., *Campylobacter* spp., and *L. monocytogenes.* However, we have combined the results together into a ‘Pathogen indicator’. The decision to use a composite ‘Pathogen indicator’ was driven strictly by statistical constraints due to low prevalence of individual organism. Attempting to model these individually resulted in quasi-complete separation and unstable odds ratios due to the sparsity of positive events. Therefore, we conceptualized the dependent variable not as a specific biological outcome, but as a ‘Pathogen indicator’. Since all three pathogens are vegetative bacteria that should be eliminated by adequate hygiene and cooking, their presence, regardless of species, serves as a robust indicator for a breakdown in the safety control system. Also, from a public health and consumer safety perspective, the presence of any of these major foodborne pathogens renders the product unsafe. By grouping them, we created an indicator that evaluates the efficacy of current intervention strategies against a set of major biological hazards.

The study identified the retail outlet as a critical hotspot for pathogen presence. Although the slaughter process inevitably introduces the initial microbial load via exposure to gut and respiratory flora (Rouger et al., 2017), it is the retail environment that facilitates amplification. Unlike the standardized conditions of industrial processing or the immediate thermal intervention in restaurants or home, retail meat is frequently subjected to handling lapses, extended display periods, and cold chain interruptions. These time–temperature fluctuations act as critical drivers for microbial proliferation (Nychas et al., 2008). Retail level has been implicated as the primary determinant of human infection risk and it can be effectively neutralized at the consumer node through rigorous hygiene and proper cooking practices (Akil and Ahmad, 2019). The public health implication is significant, as reducing contamination loads at this specific stage correlates directly with declines in foodborne illness incidence, supporting the prioritization of post-slaughter interventions (Rosenquist et al., 2003; Williams et al., 2021).

The univariate analysis initially indicated that extended pre-slaughter holding (>24 h) significantly reduced contamination odds. However, after adjusting for confounders in the multivariable model, this protective effect was most robustly defined within the 6.1–12 h window (AOR = 0.232). This refinement aligns perfectly with international best practices; the Codex Alimentarius Commission (CAC, 2011) explicitly recommends an 8–12 h feed withdrawal period to minimize the contamination of carcasses by fecal material and ingesta. This regulatory standard is supported by a previous work that have explicitly shown that a 6 to 10-h fasting period significantly reduces the likelihood of carcass contamination (Xue et al., 2020). In our study most samples were collected within 60 min of slaughter. The significant association of pathogen presence in relation to a period of 31–60 min, as opposed to longer durations, is likely due to sample distribution, where the low frequency of extended storage times limited the statistical power of the Chi-square analysis. The elevated risk associated with cotton cloth covers, compared to fly nets, is likely attributable to the physical properties of the materials. Fly nets serve as essential physical barriers, preventing the introduction of pathogens by mechanical vectors known to transport bacteria from external fecal sources (Hald et al., 2004). In contrast, porous cloths absorb moisture and organic residues, creating a damp microenvironment that facilitates microbial survival and acts as a persistent source for contamination (Todd et al., 2009). Wooden surfaces exhibited the highest frequency of pathogen presence, followed by plastic, even though the association was not statistically significant (*p* > 0.05). This trend likely reflects the inherent physical vulnerabilities of the cutting media. The porous nature of wood entraps moisture and microbes, whereas plastic surfaces eventually develop micro-fissures that facilitate bacterial adherence (Cliver, 2006). However, higher retention rates in wood do not necessarily correlate with increased transmission risk (Cliver, 2006). These physical characteristics significantly affect effective disinfection, as wooden boards and worn plastic surfaces have been shown to remain contaminated even after cleaning, supporting the recommendation for their exclusion from critical meat handling areas (Tebbutt, 1991). Work attire becomes easily contaminated during the handling of unprocessed ingredients, particularly raw meat, and serves as a source for pathogen transfer to food products if not changed at appropriate intervals (Todd et al., 2010a). In practice, this rule is often neglected, as many workers keep wearing soiled clothes instead of changing (Gemeda et al., 2024). The area beneath the fingernails traps significantly more bacteria than the fingertips or palms. And these long or untrimmed nails pose a serious risk, as these hidden germs are easily spread to meat during handling (Todd et al., 2010b). The best practices dictate that fingernails be kept short and mechanically scrubbed with a nailbrush during washing to prevent the transfer of pathogens to food products (Todd et al., 2010b). This risk associated with hand hygiene is heightened in this study, where it was observed that no butcher wore gloves during slaughtering, resulting in direct contact between hands and meat. Similarly, the presence of jewelry on hands is associated with higher pathogen retention. While this risk is frequently documented in clinical environments (Trick et al., 2003; Fagernes and Lingaas, 2011), it is equally critical in food handling settings. Accessories such as rings impede thorough washing and create protected areas for microbial survival, making the removal of hand ornaments a necessary precaution for preventing cross-contamination (Todd et al., 2010b). Higher contamination levels were observed in settings lacking glass covers or involving open-air sales, reflecting increased exposure to dust and vectors. The strong association between fly density and infectious illness confirms their role as effective mechanical vectors for spreading enteric pathogens (Collinet-Adler et al., 2015). This finding underscores the necessity of physical barriers; interventions such as installing glass covers could effectively prevent contamination by simultaneously excluding flies and reducing the deposition of airborne dust, thereby mitigating the overall microbial load in the retail environment. Pathogenic microorganisms can survive on various surfaces for extended periods, ranging from hours to days. Key foodborne bacteria, including *L. monocytogenes* and *Salmonella* spp. are notably capable of forming protective biofilms on these surfaces (Martinon et al., 2012). These biofilms, once formed within different food industry settings, become a sustained source of food contamination (Srey et al., 2013). Therefore, proper disinfection and frequent thorough cleaning are essential to eliminate microbial reservoirs and minimize the risk of contamination. Multivariable logistic regression confirmed that pre-slaughter holding periods, slaughter-to-sale intervals, outlet washing frequency, and fingernail hygiene act as independent determinants of contamination. Shorter slaughter-to-sale intervals and longer pre-slaughter holding periods emerged as significant protective factors, reflecting the critical necessity of minimizing exponential bacterial growth and ensuring effective gut clearance prior to evisceration Specifically, the finding that frequent shop cleaning and trimmed nails significantly reduced risk highlights the critical need for basic hygiene. These routine practices are essential for preventing bacterial biofilms on surfaces and thereby eliminating cross-contamination risks (Chmielewski and Frank, 2003).

At the restaurant level, the use of separate cutting boards and knives for raw and cooked meat was identified as a significant protective factor against pathogen presence. Microorganisms transfer efficiently from raw to cooked products via shared surfaces (Goh et al., 2014), while organic residues on cutting boards facilitate pathogen persistence (Kusumaningrum et al., 2004). These findings underscore the necessity of strict utensil segregation to disrupt cross-contamination pathways. Regarding temperature management, bulk cooking and hot holding showed distinct associations with contamination risk. Failure to maintain adequate hot-holding temperatures significantly increased the odds of pathogen presence, consistent with observations that inadequate thermal maintenance allows surviving microorganisms like *L. monocytogenes* to proliferate (Sun et al., 2019). Conversely, products cooked in bulk showed lower contamination risk. Large-scale cooking operations are generally forced to use more organized steps and strict rules. This organized system leads to better process control and fewer mistakes compared to small, uncontrolled batch handling (Hartantyo et al., 2023). Cross-contamination risks were evident in storage and plating practices. The use of raw vegetable garnish was associated with higher pathogen presence, corroborating studies identifying raw produce as a source for transferring pathogens to cooked meat (Omurtag et al., 2013). Furthermore, storing raw and cooked items together significantly increased contamination odds. Poor segregation and unsatisfactory storage conditions are recognized as primary contributors to microbial hazards in restaurant environments (Sani and Siow, 2014). The protective effect observed in restaurants preparing multiple chicken dishes simultaneously (AOR = 0.177) likely reflects the operational discipline inherent to high-volume kitchens. Unlike ad-hoc preparation, simultaneous multi-dish cooking typically requires structured workflows and task segregation to maintain efficiency. This organized approach minimizes the chaotic mixing of raw and cooked handling steps, thereby reducing opportunities for cross-contamination. This interpretation is reinforced by the multivariable model, which identified equipment segregation (using separate cutting boards/knives) and bulk cooking as independent protective factors. Together, these findings suggest that the rigor of organized cooking workflows and consistent utensil separation are the primary drivers of pathogen control in restaurant settings, acting as effective barriers against contamination regardless of the kitchen’s physical layout.

At the consumer level, understanding of food safety is often broad, yet many lack awareness of the specific procedures necessary to prevent contamination. This procedural gap is significant, as many fail to use separate or adequately clean utensils for raw ingredients and cooked foods, creating a high risk of cross-contamination (Redmond and Griffith, 2003). Furthermore, casual handwashing is often insufficient because pathogens stay in hard-to-clean areas, such as beneath the fingernails, thereby requiring systematic and thorough cleaning of both hands and utensils to break the contamination cycle (Todd et al., 2010b). The odds of pathogen presence were significantly lower when vegetables were added during cooking compared to when they were added post-cooking. This suggests that heat exposure during cooking may inactivate potential contaminants. Similar observations were noted indicating that mixing raw vegetables with cooked meat can promote cross-contamination (Omurtag et al., 2013). Furthermore, the odds of pathogen presence were markedly lower when separate cutting boards or knives were used for raw meat and vegetables. Comparable findings have been observed in studies, which described the use of shared boards and knives as an important contributor to microbial transfer (Kusumaningrum et al., 2004; Goh et al., 2014). Bacterial multiplication during inadequate storage (at warm ambient temperatures or slow refrigeration) significantly increases contamination risk in leftovers compared to freshly served food (Bryan, 1980). This risk is compounded when accumulated microbial growth is followed by uneven reheating, which fails to eliminate surviving pathogens. Therefore, effective process control must strictly govern all post-cooking phases, including rapid chilling and thorough reheating (Bryan, 1980). The multivariable model identified hand hygiene and utensil use as the primary determinants of contamination at the consumer level. Washing hands ‘only before handling raw meat’ was associated with significantly higher risk, whereas using separate cutting boards and knives markedly reduced it. These statistical associations confirm that preventing cross-contamination requires both strict utensil segregation and comprehensive handwashing (before and after handling) to ensure safety.

The findings from our study integrate seamlessly into existing food safety frameworks, reinforcing the model that contamination risk is not static; rather, it accumulates through a complex mix of behavioral, infrastructural, and environmental factors, most of which are indeed modifiable. The retail sector clearly emerges as the critical hotspot for microbial amplification. While the slaughter process introduces the initial microbial load, subsequent exponential growth is primarily driven by lapses in time–temperature management and insufficient environmental sanitation. Effective mitigation requires disciplined action across the entire chain. Simple, achievable interventions like, improving cleaning frequency, enforcing strict personal hygiene (including utensil segregation), and maintaining physical barriers to control vectors would substantially cut contamination loads. Ultimately, these findings underscore the necessity of strict adherence to prerequisite programs and confirm that focusing on modest, feasible interventions applied systematically yields significant and lasting improvements in microbiological safety. Achieving a reduction in foodborne illness incidence demands disciplined process control from post-slaughter handling all the way to the final consumer preparation stage.

## Data Availability

The original contributions presented in the study are included in the article/Supplementary material, further inquiries can be directed to the corresponding authors.
