# Supplementary material for: Risk factor analysis of Campylobacter spp., Listeria monocytogenes and Salmonella spp. in the chicken meat value chain
Source: Front Microbiol. 2026 Jan 12;16:1750419. doi: 10.3389/fmicb.2025.1750419 (PMC12832963; doi:10.3389/fmicb.2025.1750419)
Supplement: Supplementary file 1 [file Data_Sheet_1.docx]

**Supplementary material of article titled “Risk Factor Analysis of *Campylobacter* spp. *Listeria monocytogenes* and *Salmonella* spp. in the Chicken Meat Value Chain”**

**A. Sample Size Estimation and Design Parameters**

$$n=\frac{Z^{2}\text{ }P(1-P)}{d^{2}}$$

Where:

- n = required sample size
- Z = Z-value for desired confidence
- P = expected prevalence
- d = desired absolute precision

**ICC Formula:**

$$\rho=\frac{\sigma_{between}^{2}}{\sigma_{between}^{2}+\sigma_{within}^{2}}$$

Where:

- σ² between = variance between clusters
- σ² within = variance within clusters
- ρ (rho) = ICC

**Design Effect**

DEFF=1+(m−1) ρ

Where:

- **m** = average number of individuals per cluster
- **ρ** = intra-cluster correlation coefficient

**References**

Thrusfield, M. (2018). Veterinary Epidemiology, 4th Edition. Wiley Blackwell.

**B. Definitions of variable used in the risk factor analysis**

**1. Clean costumes:** Use of dedicated, intact protective outer clothing that is visibly free

from blood spatters, feathers, and organic debris. The clothing is worn over

street clothes and is restricted to the food handling area to prevent cross-

contamination.

**2. Unclean costumes:** Absence of dedicated clothes or the use of visibly soiled or

damaged clothing containing deposits of blood, feathers, or meat debris. This

category includes practices where the handler wipes soiled hands or knives

directly onto their clothing or wears the clothes outside the designated sanitary

zone.

**3. Fly Density:**

- **Low Density:** ≤ 10 fly observed in the immediate vicinity of the meat within

a 1-minute observation period.

- **High Density:** >10 flies observed hovering or landing on the meat/surfaces

within a 1-minute observation period.

**4. Hot Holding:**

- **Hot Holding:** Maintaining cooked chicken meat at a core temperature of **≥**

60°C (140°F) continuously until served.

- **No Hot Holding:** Holding cooked meat at ambient temperature (danger

zone: (5°C – 60°C) for > 1 hours.

**5. Bulk Cooking:** The preparation of meat in quantities > 3 kg per batch.

.

**C. Details of Primers used for molecular confirmation of *Campylobacter* spp., *Listeria***

***monocytogenes* and *Salmonella* spp.**

| **Details of Primers used for molecular confirmation** | | | | | |
| --- | --- | --- | --- | --- | --- |
| **Sl. No** | **Pathogen Indicator** | **Target gene** | **Primer sequence** | **Base pair** | **Reference** |
| **1** | ***Campylobacter* spp.** | *16S rRNA* | **F-** GGA TGA CAC TTT TCG GAG C | 816bp | (Linton *et al.,* (1996) |
|  |  |  | **R-** CAT TGT AGC ACG TGT GTC |  |  |
| **2** | ***Listeria* *monocytogenes*** | *prs* | **F-** AGC TGA AGA GAT TGC GAA AGA | 844bp | (Rawool *et al.,*  2016) |
|  |  |  | **R-** TTC ACC AAG AAG AGC TGC AA |  |  |
|  |  | *isp* | **F-** TGC AGC GAA TGC TCT TAG TG | 713bp |  |
|  |  |  | **R-** AGC CAA GCA CGG CTA CTT TA |  |  |
| **3** | ***Salmonella* spp.** | *invA* | **F**:ACAGTGCTCGTTTACGAC  CTGAAT | 284 bp | (Chiu and Ou, 1996) |
|  |  |  | **R**:AGACGACTGGTACTGATC  GATAAT |  |  |

References

Linton, D., Owen, R. J., and Stanley, J. (1996). Rapid identification by PCR of the genus *Campylobacter* and of five *Campylobacter* species enteropathogenic for man and animals. *Res. Microbiol.* 147, 707–718. doi: 10.1016/s0923-2508(97)85118-2.

Rawool, D. B., Doijad, S. P., Poharkar, K. V., Negi, M., Kale, S. B., Malik, S. V. S., Kurkure, N.V., Chakraborty, T. and Barbuddhe, S.B. (2016). A multiplex PCR for detection of *Listeria monocytogenes* and its lineages. *J. Microbiol. Methods* 130, 144–147. doi: 10.1016/j.mimet.2016.09.015

Chiu CH & Ou JT (1996). Rapid identification of *Salmonella* serovars in feces by specific detection of virulence genes, *invA* and *spvC*, using an enrichment broth culture–multiplex PCR combination assay. *Journal of Clinical Microbiology*, 34(10), 2619–2622. doi.org/10.1128/jcm.34.10.2619-2622.1996
